# Novel technique of repairing right partial anomalous pulmonary venous connection with intact atrial septum using in situ interatrial septum as a flap in a 68-year-old-woman: a case report

**DOI:** 10.1186/s13019-020-01313-w

**Published:** 2020-09-25

**Authors:** Atsushi Morishita, Ikuo Hagino, Hideyuki Tomioka, Seiichiro Katahira, Takeshi Hoshino, Kazuhiko Hanzawa

**Affiliations:** 1 Department of Cardiovascular Surgery, Numata Neurosurgery Heart-Disease Hospital, 8 Sakae-cho, Numata, 378-0014 Japan; 2 Department of Cardiovascular Surgery, Chiba Children’s Hospital, Chiba, Japan; 3 Department of Cardiovascular Surgery, Tokyo Women’s Medical University Yachio Medical Center, Yachio, Japan; 4 Division of Health Administration, Hamakawasaki Operation Center, Toshiba Human Asset Service Corporation, Kawasaki, Japan; 5 Department of Anesthesiology, Minami Machida Hospital, Machida, Japan; 6 Department of Advanced Treatment and Prevention for Vascular Disease and Embolism, Niigata University Graduate School of Medical and Dental Sciences, Niigata, Japan

**Keywords:** Partial anomalous pulmonary venous connection, Intact atrial septum, Elderly, Interatrial septum flap

## Abstract

**Background:**

Partial anomalous pulmonary venous connection draining into the right atrium with an intact atrial septum is a very rare clinical entity in the adult population. Partial anomalous pulmonary venous connection must be suspected as a differential diagnosis when the cause of right heart enlargement and pulmonary artery hypertension is unknown.

**Case presentation:**

This study describes the surgical case of an isolated right partial anomalous pulmonary venous connection to the right atrium in a 68-year-old woman, who underwent tricuspid ring annuloplasty and right-sided maze procedure simultaneously. She had complaints of gradually progressing dyspnea on exertion. However, a diagnosis could not be established despite consultations at multiple hospitals for over a year. Right heart catheterization revealed severe pulmonary artery hypertension with a mean pulmonary artery pressure of 46 mmHg, step-up phenomenon of oxygen saturation at the mid-level of the right atrium with a pulmonary-to-systemic blood flow ratio of 2.4, and a pulmonary vascular resistance of 3.1 Wood Units. As medical treatment with pulmonary artery vasodilator therapy did not improve her symptoms, she underwent surgical repair. An atrial septal defect was created surgically with a curvilinear tongue-shaped cut. The right anomalous pulmonary veins were rerouted through the surgically created atrial septal defect into the left atrium with a baffle comprised of the interatrial septum flap, kept in continuity with the anterior margin and sutured while mobilizing the enlarged right atrium. The patient had an uneventful postoperative course and remains asymptomatic.

**Conclusions:**

The described surgical technique could be considered an effective alternative for patients undergoing surgical repair for a partial anomalous pulmonary venous connection isolated to the right atrium. The indication for surgery must be judged on a case-by-case basis in these patients with prevalent systemic-to-pulmonary shunting.

## Background

Partial anomalous pulmonary venous connection (PAPVC) is a rare congenital anomaly, and it is seldom found in the adult population [1, 2]. Furthermore, PAPVC draining solely into the right atrium (RA) with an intact atrial septum (i.e., an isolated right PAPVC to the RA) is a very rare entity [3, 4]. Adult patients with PAPVC often suffer from nonspecific complaints such as fatigue, dyspnea, and palpitations, which can remain undiagnosed, as seen in the present case report. It is crucial to suspect PAPVC as a differential diagnosis in adult patients in whom the cause of right heart enlargement and pulmonary artery hypertension is unknown. This report describes the case of a 68-year-old woman with an isolated right PAPVC that was treated surgically; the right anomalous pulmonary veins were rerouted into the left atrium (LA) using a baffle, comprised of the flap of the interatrial septum, through the surgically created atrial septal defect (ASD).

## Case presentation

**Fig. 1:**
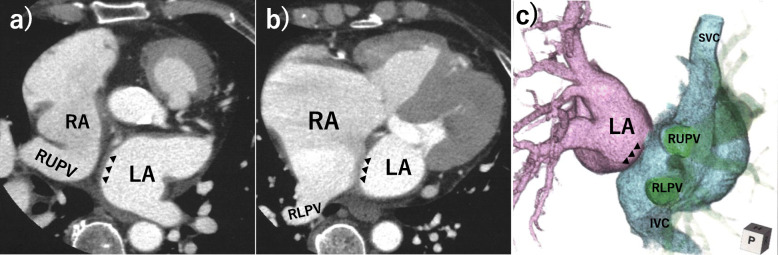
Preoperative multidetector computed tomography angiography. Preoperative multidetector computed tomography angiography demonstrating the anomalous drainage of both the right upper and lower pulmonary veins into the right atrium. The intact atrial septum (black arrowheads) is small and the plane is displaced leftward. Axial images (**a**, **b**) and three-dimensional reconstruction (**c**) are presented. LA = left atrium, RA = right atrium, RUPV = right upper pulmonary. Vein, RLPV = right lower pulmonary vein, SVC = superior vena cava, IVC = inferior vena cava

A 68-year-old woman complaining of dyspnea on exertion was admitted to our hospital. Her medical history included hypertension for several years, for which she had been treated. Despite consultations at multiple hospitals for over a year, a diagnosis could not be established for her gradually worsening symptoms. On admission, her blood pressure was 132/62 mmHg. Her pulse rate was 72 beats/min and regular. A grade II/VI midsystolic heart murmur was evident on auscultation in the second intercostal space in the parasternal plane. A chest radiograph revealed moderate cardiomegaly with a cardiothoracic ratio of 65% and an increased pulmonary vascular shadow. An electrocardiogram (ECG) showed sinus rhythm with a right bundle branch block pattern. Laboratory evaluations revealed no abnormalities. Transthoracic echocardiography (TTE) revealed a normal function of the left ventricle and severe dilatation of the right cardiac chambers associated with diastolic ventricular septal flattening. Severe tricuspid regurgitation and absence of visible interatrial communication was also observed. Transesophageal echocardiography (TEE) clearly demonstrated the draining of both the right upper and right lower pulmonary veins into the RA with an intact atrial septum. ECG-gated multidetector computed tomography (MDCT) revealed anomalous drainage of both the right upper and right lower pulmonary veins into the RA (Fig. 1). In addition, the positional relationship between the superior vena cava (SVC), inferior vena cava (IVC), and orifices of the anomalous pulmonary veins, with the exception of the atrial septum, was consistent with the normal anatomy. Furthermore, the size of the intact atrial septum was diminished, and its plane was displaced leftward. No further congenital cardiovascular anomalies were observed. No significant coronary stenosis was identified by coronary angiography. Right heart catheterization (RHC) concurrently revealed severe pulmonary artery hypertension with a mean pulmonary artery pressure (MPAP) of 46 mmHg, step-up of oxygen saturation at the mid-level of the RA with a pulmonary-to-systemic blood flow ratio (Qp/Qs) of 2.4, a pulmonary-to-systemic vascular resistance ratio (Rp/Rs) of 0.11, a pulmonary-to-systemic artery systolic pressure ratio (Pp/Ps) of 0.42, and a pulmonary vascular resistance (PVR) of 3.1 Wood Units. Oxygen inhalation during RHC decreased MPAP from 46 to 41 mmHg, resulting in no significant changes. After a comprehensive evaluation, PAPVC involving the right pulmonary veins draining into the RA with an intact atrial septum was diagnosed. A patient took 62.5 mg of Bosentan twice a day at her discretion for 6 months. Since there was no improvement in clinical symptoms, the patient underwent surgical repair after providing written informed consent.

**Fig. 2:**
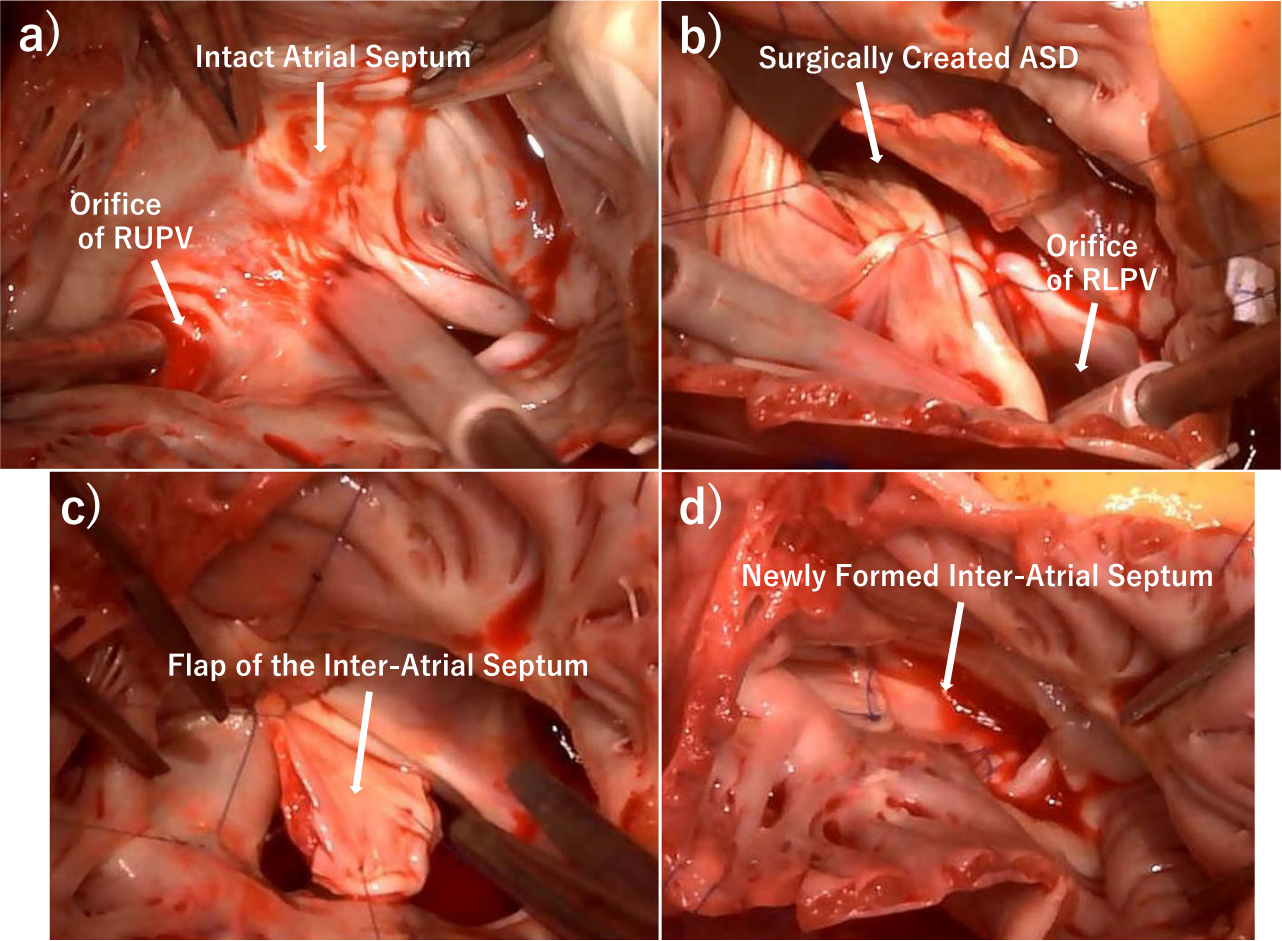
Intraoperative photographs. Intraoperative photographs demonstrating an intact atrial septum (**a**), the reconstructed endocardial layer between the LA and the RA (**b**), the flap of the inter-atrial septum (**c**), and the newly formed inter-atrial septum (**d**). RUPV = right upper pulmonary vein, ASD = atrial septal defect, RLPV = right lower pulmonary vein

**Fig. 3:**
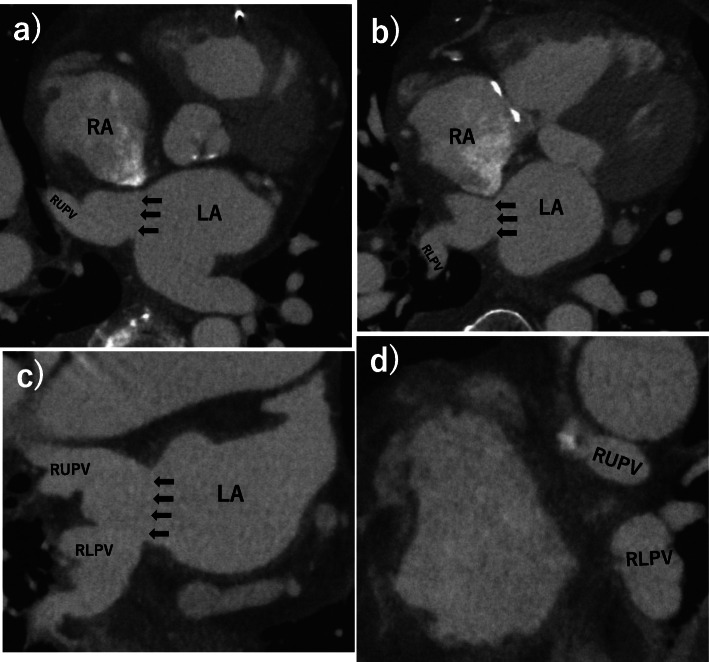
Postoperative multidetector computed tomography angiography. Postoperative multidetector computed tomography angiography demonstrating the intra-atrial baffle that show neither the pulmonary vein stenosis nor vena caval obstruction. Surgically created atrial septal defect in an appropriate size (black arrow) was recognized. Axial (**a**, **b**), coronal oblique (**c**), and sagittal oblique (**d**) images are presented. LA = left atrium, RA = right atrium, RUPV = right upper pulmonary vein, RLPV = right lower pulmonary vein

A median sternotomy was performed, and the position of the anomalous right lower pulmonary vein was identified to be adjacent to the IVC orifice. The IVC was cannulated through the right femoral vein in order to achieve satisfactory exposure of the RA. Through right atriotomy, an anomalous connection of both the right upper and right lower pulmonary veins to the RA with an intact atrial septum was identified (Fig. 2a). The posterior side of coronary sinus formed a steep bank and led to the IVC. Initially, the maze procedure in the RA was performed by means of bipolar radiofrequency ablation and cryoablation. Subsequently, ASD was created surgically; the incision extended posteriorly from the superior margin of the fossa ovalis toward the inferior margin of the septum secundum showing a curvilinear tongue-shaped cut. The flap of the interatrial septum, which was kept in continuity with the anterior margin and covered an area of 15 mm^2^, was completed. For the purpose of preventing thrombus formation in the exposed muscle and for approximating the orifices of the right lower anomalous pulmonary vein to the excised atrial septum as far as possible, the endocardial layer between the LA and the RA was reconstructed with 5–0 monofilament running sutures while maintaining an extroverted configuration (Fig. 2b). The right anomalous pulmonary veins were rerouted with the flap of the interatrial septum baffle through the surgically created ASD into the LA using 5–0 monofilament continuous sutures, mobilizing the RA. The anastomotic portion of the superior, inferior, and posterior margin of the flap were formed by the posterior wall of the SVC-RA junction, the posterior wall of the IVC-RA junction, and the right lateral wall just above the right pulmonary vein orifices, respectively (Fig. 2c, d). Finally, tricuspid annuloplasty was performed with a 28-mm Carpentier-Edwards PhysioTricuspid ring (Edwards LifeSciences, Irvine, CA) for annular enlargement. Weaning from the cardiopulmonary bypass was successful. The patient had an uneventful postoperative course without undesirable complications related to sinus node dysfunction and residual intracardiac shunt. Postoperative ECG-gated MDCT demonstrated that the intra-atrial baffle showed neither a pulmonary vein stenosis nor vena cava obstruction (Fig. 3). The patient was discharged from the hospital on postoperative day 27 without the use of Bosentan. At follow-up 1 year later, the patient remained asymptomatic with normal echocardiography.

## Discussion and conclusions

PAPVC is a rare congenital anomaly with a prevalence in the adult population of 0.1 and 0.31% when detected by MDCT and magnetic resonance imaging, respectively [1, 2]. In the adult population, PAPVCs originating from the pulmonary lobes are distributed in the following order of frequency: left upper lobe in 47%, right upper lobe in 38%, right lower lobe in 13%, and left lower lobe in 2% of cases; PAPVC originating from the right upper lobe has a propensity to be moderately associated with sinus venous ASD [1]. Isolated PAPVC to the RA can occur in approximately 5% of the adult and pediatric population with PAPVC to the right side of the heart, as reported by Gustafson et al. [3]. Isolated PAPVC to the RA in adults was absent in a retrospective review, as reported by Majdalany et al. [4]. TTE may not always detect PAPVC due to its limited acoustic window. However, TEE might be a valuable adjunct in terms of the accurate delineation of congenital malformations, with the exclusion of difficulty in identifying left PAPVC. MDCT has been an effective imaging modality that is able to provide reliable information for confirming the diagnosis and making an efficient surgical plan with regards to complex pulmonary venous anatomy [5].

According to the 2015 European Society of Cardiology and the European Respiratory Society guidelines for the diagnosis and treatment of pulmonary hypertension, surgery may be considered in patients with prevalent systemic-to-pulmonary shunting. The correction of congenital heart disease is recommended if PVR is less than 2.3 Wood Units but not recommended if the PVR is more than 4.6 Wood Units [6]. The 2018 American Heart Association and American College of Cardiology guidelines for the management of adults with congenital heart disease state that surgical repair is recommended for patients with PAPVC under certain conditions, which involved a Qp/Qs of more than 1.5, a Rp/Rs of less than one third, and a Pp/Ps of less than 0.5 [7]. Based on the criteria for shunt closure and clinical presentation, surgical intervention was deemed appropriate in the present patient. In patients with pulmonary artery hypertension associated with congenital heart disease which include Eisenmenger syndrome and high surgical risks due to multiple comorbidities, surgery is contraindicated. In this situation, medical treatment with pulmonary artery vasodilator therapies had been proposed, as it has been suggested that the use of Bosentan, phosphodiesterase inhibitors, and intravenous epoprostenol shows favorable functional and hemodynamic improvements [6]. Dimopoulos et al. suggests that the “treat-and-repair” concept, which combines advanced pulmonary artery vasodilator therapies with surgical repair of the defect, may be considered as an alternative approach in highly selected patients, but available data is still limited [8]. In the case that a patient is refractory to medical treatment, heart-lung or lung translation with heart surgery may be the only curative option.

If the anomalous pulmonary veins are connected to the RA only, the blood stream of the anomalous pulmonary veins can be diverted through the ASD and into the LA with the intra-atrial baffle, with less likelihood of rhythm disturbances. Given that the intact atrial septum was halfway between the right and left pulmonary venous orifices, and the right anomalous pulmonary veins maintained their normal anatomical positional relationship with the adjacent structures in this patient, an extensive baffle was necessary to reroute from the orifices of the right pulmonary veins to the surgically created ASD. Since the pressure in the newly formed LA was predicted to increase postoperatively, it was important to choose an appropriate size and design for the intra-atrial baffle. A convex bowing of the newly formed inter-atrial septum towards the RA, particularly when its size is large, may result in stenosis or obstruction of the SVC or IVC. Regarding the intra-atrial baffle, a polytetrafluoroethylene patch or untreated autologous pericardial patch are generally used [9]. However, we have adopted the repositioning of the flap of the interatrial septum so as to take advantage of the antithrombogenicity, ensuring a non-redundant baffle and avoiding potential baffle shrinkage, without removing a portion of the atrial septum or using other types of intra-atrial patches. This technique mostly depends on the size of the excised atrial septum and the positional relationship among the inter-atrial septum, the right anomalous pulmonary vein orifices, the SVC, and the IVC. In the present case, the length of the atrial flap was shorter than the distance between the right upper pulmonary vein and right lower pulmonary vein orifices. In order to maintain the strength of the suture, minimize the risk of tissue disruption, and complete the baffle with the interatrial septum flap alone, the flap was reinforced with suture plications of the enlarged RA that was mobilized securely in the circumference of the intracardiac anastomotic site. This manipulation helps to prevent potential complications in the future. It was thought to be of the utmost importance that the endocardial layer between the LA and the RA was reconstructed while maintaining an extroverted configuration to prevent thrombus formation of the excised end of the atrial septum. Moreover, this additional manipulation plays an important role in allowing a shorter and straightforward pathway between the orifice of right lower anomalous pulmonary vein and the excised atrial septum. This careful placement of the intended suture bites would attribute to the relief of unbalanced turbulent blood flow into the LA during the diversion of the anomalous pulmonary venous connection.

The selection of the flap from the interatrial septum as an intra-atrial baffle, redirected from the orifices of right anomalous pulmonary veins to the surgically created ASD, could be considered as an effective alternative for patients undergoing surgical repair in an isolated PAPVC to the RA. The present case highlights the importance of high suspicion and accurate diagnosis of PAPVC in adult patients who remain undiagnosed. Additionally, the early detection of PAPVC undoubtedly would lead to the subsequent surgical treatment as a conceivable therapeutic option.

## Data Availability

The datasets supporting the conclusions of this article are included within the article.

## Abbreviations

PAPVC: Partial anomalous pulmonary venous connection
RA: Right atrium
LA: Left atrium
TTE: Transthoracic echocardiography
TEE: Transesophageal echocardiography
MDCT: Multidetector computed tomography angiography
SVC: Superior vena cava
IVC: Inferior vena cava
RHC: Right heart catheterization
ASD: Atrial septal defect
ECG: Electrocardiogram
Qp/Qs: Pulmonary-to-systemic blood flow ratio
Rp/Rs: Pulmonary-to-systemic vascular resistance ratio
Pp/Ps: Pulmonary-to-systemic artery systolic pressure ratio
PVR: Pulmonary vascular resistance
